# Knowledge and Awareness of Orthodontic Emergencies Among Dental Students and Dentists: A Cross‐Sectional Study

**DOI:** 10.1111/eje.70256

**Published:** 2026-07-24

**Authors:** Kübra Arslan Çarpar, Tuğba Yılmaz

**Affiliations:** ^1^ Department of Orthodontics, Faculty of Dentistry Mersin University Mersin Turkiye

**Keywords:** awareness, knowledge level, orthodontic emergency, perceived competence

## Abstract

**Objectives:**

This study aimed to evaluate the knowledge levels of dental students and graduate dentists regarding orthodontic emergencies and to identify factors independently associated with knowledge.

**Methods:**

This cross‐sectional descriptive survey included fourth‐ and fifth‐year dental students and graduate dentists from dental schools across Türkiye. Data were collected via an online questionnaire with three sections: demographic characteristics, knowledge regarding orthodontic emergencies (0–10 points), and awareness and perceived competence (5–25 points). Data were analysed using parametric and nonparametric statistical tests. Factors independently associated with knowledge level were examined using multiple linear regression analysis.

**Results:**

The mean knowledge score of the 460 participants was 7.08 ± 2.31. Participants performed better in basic clinical management but showed significantly lower performance in clinical prioritization and emergency intervention scenarios (*p* < 0.001). A positive and statistically significant correlation was found between total knowledge score and awareness score (*ρ* = 0.295, *p* < 0.001). Multiple linear regression analysis showed that awareness score showed the strongest association with knowledge score in the adjusted regression model (*β* = 0.302, *p* < 0.001). Graduate dentists had significantly higher knowledge scores than students, whereas male gender was negatively associated with knowledge score.

**Conclusion:**

Although dentists and dental students demonstrated adequate basic management knowledge regarding orthodontic emergencies, they showed important deficiencies in clinical prioritization and decision‐making. These findings may identify areas for further curricular evaluation, particularly regarding clinical prioritization and emergency decision‐making. Scenario‐based and practice‐oriented approaches may be considered as evidence‐informed options for future educational development; however, their effectiveness should be evaluated in prospective intervention studies.

**Abbreviations**
NSAIDs: Nonsteroidal Anti‐Inflammatory Drugs
OSCE: Objective Structured Clinical Examination
TPA: Transpalatal Arch

## 1 Introduction

Orthodontic treatments are increasingly common among both pediatric and adult patients, and the likelihood of encountering orthodontic treatment‐related emergencies is rising accordingly. Situations such as protruding wire, bracket or retainer breakage, soft tissue trauma caused by appliances, and pain associated with orthodontic appliances can cause serious discomfort for patients and often occur unexpectedly. Such emergencies are frequently managed by general dentists, who are the first point of contact when direct access to orthodontic specialists is not possible.

Effective management of orthodontic emergencies depends not only on technical skills but also on appropriate clinical prioritization and decision‐making. The literature reports that dentists can perform basic interventions in orthodontic emergencies but struggle to determine which situations require urgent intervention [1]. Incorrect or delayed management can lead to prolonged pain, increased soft tissue damage, and a negative impact on treatment.

Orthodontic courses in dental undergraduate education are generally confined to theoretical knowledge and insufficient clinical observation, with insufficient emphasis on practical, scenario‐based training for orthodontic emergencies. This may cause students and recent graduates to feel inadequate in such cases. Scenario‐based learning approaches have been shown to significantly improve clinical decision‐making skills [2].

In addition to knowledge, perceived competence may be relevant to how individuals approach clinical situations. However, the present study assessed awareness and perceived competence rather than clinical self‐efficacy using a validated self‐efficacy instrument [3]. Studies in health professions education have reported associations between self‐efficacy and clinical decision‐making or patient management outcomes [4, 5, 6]. However, self‐efficacy was not directly measured in the present study; the questionnaire assessed awareness and perceived competence.

The current literature contains few studies assessing the knowledge, awareness, and perceived competence of dental students and graduate dentists in Türkiye regarding orthodontic emergencies. Most existing studies are based on a single institution and do not simultaneously address student–graduate comparisons and cognitive level analysis. Given this gap, the present study aimed to evaluate the knowledge and awareness levels of dental students and graduate dentists regarding orthodontic emergencies, to identify performance differences by cognitive level, and to examine factors independently associated with knowledge level. The findings may help inform future dental curriculum evaluation and the design of continuing professional education programs.

## 2 Methods

This study is a cross‐sectional, descriptive survey designed to assess the knowledge and awareness of orthodontic emergencies among dental students and graduate dentists. The study protocol received approval from the Mersin University Clinical Research Ethics Committee on October 1, 2025, with decision number 2025/1079. Informed consent was obtained online from all participants. The target population of the study consisted of dentists throughout Türkiye, specialists and research assistants working in various dental specialties, and fourth‐ and fifth‐year dental students.

The data collection tool was created using Google Forms and was administered online. The survey questions were prepared by a research assistant and an orthodontist in accordance with the literature; another research assistant and an orthodontist reviewed them for content validity.

The survey is divided into three sections.

1. *Demographic information*: This section asked participants about their age, gender, education level, the university they graduated from or are currently attending, and their orthodontic treatment status.
2. *Knowledge level*: The level of understanding regarding orthodontic emergencies was evaluated using 10 multiple‐choice questions covering wire punctures, bracket or retainer breakage, orthodontic appliance‐related pain, orthodontic mini‐screw loosening, soft tissue trauma caused by fixed appliances, and approaches to medication use. Each correct answer was awarded one point, and the total knowledge score was calculated on a scale of 0–10. A higher score indicates a greater level of knowledge.
  The knowledge items were independently classified by two researchers before analysis based on the cognitive level they assessed, and the final classification was reached by consensus. Items were categorized into three groups: (i) conceptual/diagnostic level (S1, S3, S9), (ii) basic clinical management (S2, S4, S5), and (iii) clinical prioritization and situations requiring urgent intervention (S6, S7, S8, S10). This classification was grounded in the complexity of clinical decision‐making and was used for performance comparisons across cognitive levels. The knowledge items were independently classified by two researchers prior to analysis according to the cognitive level they measured and finalized by consensus. Items were evaluated under three categories: (i) conceptual/diagnostic level (S1, S3, S9), (ii) basic clinical management (S2, S4, S5), and (iii) clinical prioritization and situations requiring urgent intervention (S6, S7, S8, S10). This classification was based on Bloom's cognitive taxonomy, which categorizes learning outcomes from lower‐order processes to higher‐order cognitive skills such as analysis and evaluation [7].
3. *Awareness and perceived competence*: Participants' awareness and perceived competence regarding orthodontic emergencies were assessed using a 5‐item Likert‐type scale. Response options ranged from strongly disagree to strongly agree on a five‐point scale. The total awareness score ranged from 5 to 25. A higher score indicates greater perceived competence. The internal consistency of the scale was assessed using Cronbach's alpha coefficient.

Due to uncertainty about the population size, the study used convenience sampling. The sample size was calculated using the formula *n* = *Z* ^2^ *x p*(1−*p*)/*e* ^2^ for prevalence estimation. Assuming a 95% confidence level (*Z* = 1.96), *p* = 0.50, and margin of error (*e*) = 0.05, the minimum sample size was determined to be 385. Considering the possibility of non‐response and missing data, 15% was added to this number, and the target sample size was set at 440 people. The sample size was calculated based on the prevalence estimate, and the final sample (*n* = 460) also provides sufficient power for multiple linear regression and group comparisons.

The survey link was distributed through email, professional social media groups, and other professional communication platforms. No public announcement was made. Participation was completely voluntary, and no personal identifying information was collected.

### 2.1 Statistical Analysis

Data analysis was conducted using IBM SPSS Statistics 27.0 (IBM Corp., Armonk, NY, USA) software. Descriptive statistics are reported as mean, standard deviation, and percentage values. The normality of data distribution was evaluated by examining skewness and kurtosis values. Since these values fell within the range of −1 to +1, the data were considered normally distributed, and parametric tests were deemed suitable. The total knowledge score was treated as a continuous variable, and parametric tests were used because the assumption of normality was satisfied. The Spearman rank correlation coefficient (*ρ*) was employed in correlation analyses since the Likert‐type awareness items were ordinal. The threshold for statistical significance was set at *p* < 0.05 for all analyses.

The independent samples *t*‐test was used to compare two groups. Welch's correction was applied when assumption of equal variances was violated. One‐way analysis of variance (ANOVA) was used for comparisons involving three or more groups; when significant results were found, pairwise comparisons were conducted using the Tukey multiple comparison test. Effect sizes were reported using Cohen's *d* for two‐group comparisons and eta‐squared (*η* ^2^) for multiple group comparisons.

Multiple linear regression analysis was conducted to identify factors independently associated with knowledge level. Because the occupational status variable was categorical, dummy variables were created and included in the model, with students serving as the reference group. The issue of multicollinearity was evaluated using the variance inflation factor (VIF) and condition index values.

After classifying the knowledge items by cognitive levels, the participants' performance in the three categories (conceptual/recognition, basic management, and clinical priority) was assessed in a repeated measures design and compared using the Friedman test. When significance was found, the Bonferroni‐corrected Wilcoxon matched‐pairs signed‐rank test was used. Effect sizes were reported with Kendall's *W* and the *r* coefficient.

## 3 Results

### 3.1 Participant Characteristics

A total of 484 responses were collected for the study. Nineteen participants were identified as orthodontists, and five forms with missing or incorrect data were excluded, resulting in a final sample of 460 participants.

The sociodemographic and professional characteristics of the participants are shown in Table 1. Most participants were women (63.0%) and aged 20–25 (57.4%). About half of the sample were graduates or students at Mersin University.

**TABLE 1:** Sociodemographic and professional characteristics of participants.

Variable	*n*	%
Gender		
Female	290	63.0
Male	170	37.0
Occupational status		
4th‐year dental student	173	37.6
5th‐year dental student	98	21.3
General dentist	145	31.5
Specialist/research assistant	44	9.6
Age group (years)		
20–25	264	57.4
26–30	119	25.9
31–35	28	6.1
36 years and above	49	10.7
Orthodontic treatment practice		
Yes	19	4.1
No	441	95.9
Type of orthodontic treatment^a^		
Removable appliance	14	73.7
Clear aligner	11	57.9
Fixed orthodontic treatment	10	52.6
Functional appliance treatment	6	31.6
Lingual orthodontics	1	5.3
Orthognathic surgery	0	0

^a^ Multiple responses were allowed; percentages were calculated among those who answered “Yes” (*n* = 19).

When analyzing the professional distribution, most participants were 4th and 5th‐year students and practicing dentists. A total of 4.1% of participants reported actively providing orthodontic treatment. Among those performing orthodontic procedures, the most commonly used method was removable appliances, followed by clear aligners and fixed orthodontic treatments.

### 3.2 Knowledge Level

#### 3.2.1 Overall Knowledge Score

The participants' average total knowledge score was 7.08 ± 2.31 (median: 7.5; IQR: 3; range: 0–10).

#### 3.2.2 Question‐Based Performance Analysis

The lowest correct response rates for knowledge questions were observed in S1 (58.3%) and S6 (58.0%), while the highest was in S4 (92.2%) (Table 2).

**TABLE 2:** Frequency and percentage distribution of responses to orthodontic emergency information items.

Q	Option	*n*	%
1. Which of the following is not considered an orthodontic emergency?	Gingival bleeding^a^	268	58.3
	Retainer breakage	56	12.2
	Protruding wire	51	11.1
	Bracket detachment	48	10.4
	I don't know	37	8.0
2. If a wire is poking into the patient's cheek, what should be done to provide temporary relief?	Apply orthodontic wax^a^	292	63.5
	Cut the wire	138	30.0
	I don't know	20	4.3
	Start antibiotics	7	1.5
	Remove the brackets	3	0.7
3. Which of the following is not recommended for pain caused by orthodontic appliances?	Antibiotic therapy^a^	344	74.8
	I don't know	38	8.3
	Paracetamol	35	7.6
	Orthodontic wax	23	5.0
	Cold compress	20	4.3
4. What would you recommend to a patient whose bracket and wire have become loose?	Consult their orthodontist^a^	424	92.2
	Cut the wire	15	3.3
	I don't know	10	2.2
	Discontinue orthodontic treatment	7	1.5
	Consult another dentist	4	0.9
5. What should be done if soft tissue trauma occurs due to displacement of an orthodontic wire during fixed treatment?	Cut the wire end and apply orthodontic wax^a^	372	80.9
	Remove the wire	36	7.8
	I don't know	36	7.8
	Observation only	13	2.8
	Discontinue orthodontic treatment	3	0.7
6. If a patient's retainer wire is broken and causing pain, what is the first step in management?	Remove the retainer and refer the patient to an orthodontist^a^	276	60.0
	Fabricate a new retainer	107	23.3
	I don't know	47	10.2
	Keep the retainer in place	19	4.1
	Discontinue retainer use	11	2.4
7. What should be done if an orthodontic miniscrew becomes loose?	Provide hygiene instructions and refer to an orthodontist^a^	325	70.7
	Tighten the screw	61	13.3
	I don't know	45	9.8
	Remove the screw	26	5.7
	No intervention required	3	0.7
8. A patient reports severe oral ulcers while wearing fixed orthodontic appliances. What is the most appropriate approach?	Recommend a topical corticosteroid and orthodontic wax^a^	321	69.8
	I don't know	57	12.4
	Remove the appliance	49	10.7
	Prescribe antibiotics	28	6.1
	Discontinue orthodontic treatment	5	1.1
9. What is the most commonly recommended medication for pain control in the early stage of orthodontic treatment?	Paracetamol^a^	348	75.7
	Ibuprofen	50	10.9
	I don't know	46	10.0
	Naproxen	12	2.6
	Aspirin	4	0.9
10. A 16‐year‐old patient presents with pain caused by a transpalatal arch (TPA) embedded in the palate after displacement from the bands. What is the most appropriate emergency management?	Apply local anaesthesia, carefully remove the TPA, and apply an antiseptic^a^	298	64.8
	Do not intervene and refer the patient to an orthodontist	72	15.7
	I don't know	53	11.5
	Pull the TPA forcefully and remove it	25	5.4
	Leave the TPA in place and prescribe antibiotics	12	2.6

^a^ The correct answer.

When the distribution of incorrect answers was examined, errors were concentrated in specific clinical patterns. In question S6, 23.3% of participants suggested making a new retainer directly instead of pursuing acute management. In question S2, 30.0% preferred cutting the wire rather than using a temporary relief approach. In scenario S10, 15.7% of participants indicated that no intervention should be performed.

No significant difference was observed between professional groups for questions S1 and S6 (*p* > 0.05).

#### 3.2.3 Performance by Cognitive Levels

The knowledge items were assessed across three cognitive categories. The Friedman test results indicated a significant difference between the categories (*χ* ^2^(2) = 52.364; *p* < 0.001; Kendall's *W* = 0.057).

Based on the average rankings, the highest performance was noted in basic clinical management questions, while the lowest was seen in clinical prioritization and emergency intervention questions.

In Wilcoxon analyses with Bonferroni correction:

- Basic clinical management > conceptual questions (*Z* = −6.110; *p* < 0.001; *r* = 0.28).
- Basic clinical management>clinical prioritization and emergency intervention (*Z* = −9.112; *p* < 0.001; *r* = 0.42).
- Conceptual > clinical prioritization and emergency intervention (*Z* = −2.522; *p* = 0.012; *r* = 0.12).

(Post‐Bonferroni significance level *p* < 0.017).

Effect sizes are small.

### 3.3 Comparison of Knowledge Scores Based on Demographic and Professional Factors

Knowledge scores were compared based on demographic and professional variables, and the results are shown in Table 3. A statistically significant difference in knowledge scores was observed between professional groups (*F*(2, 457) = 9.77; *p* < 0.001; *η* ^2^ = 0.04). According to the post hoc analysis, students' knowledge scores were significantly lower than those of both general dentists and specialists (*p* < 0.05). No significant difference was found between general dentists and specialists.

**TABLE 3:** Comparison of knowledge scores according to demographic and professional variables.

Variable	Group	*n*	Mean ± SD	Test	*p*	Effect size
Occupational status	Student	271	6.70 ± 2.35	*F*(2, 457) = 9.77	< 0.001	*η* ^2^ = 0.04
	General dentist	145	7.57 ± 2.19			
	Specialist	44	7.86 ± 1.94			
Academic year (students only)	4th year	173	6.39 ± 2.57	*t* = −3.27	0.001	*d* = 0.37
	5th year	98	7.27 ± 1.80			
Gender	Female	290	7.40 ± 2.22	*t* = 3.89	< 0.001	*d* = 0.37
	Male	170	6.54 ± 2.34			
Orthodontic treatment practice	Yes	19	8.42 ± 1.22	*t* = 4.64	< 0.001	*d* = 0.61
	No	441	7.03 ± 2.33			

*Note: F*: One‐way analysis of variance (ANOVA); *t*: independent samples *t*‐test. Effect sizes are presented as eta squared (*η* ^2^) for ANOVA and Cohen's *d* for *t*‐tests.

The analysis of students revealed that fifth‐year students' knowledge scores were significantly higher than those of fourth‐year students (*t* = −3.27; *p* = 0.002; Cohen's *d* = 0.37).

In the gender comparison, female participants' knowledge scores were higher than those of males (*t*(458) = 3.89; *p* < 0.001; Cohen's *d* = 0.37).

Participants who actively provided orthodontic treatment scored significantly higher in knowledge than those who did not (Welch *t* = 4.64; *p* < 0.001; Cohen's *d* = 0.61). Similarly, when students were excluded and only graduate participants were analysed, those who actively provided orthodontic treatment also had higher knowledge scores (Welch *t* = 2.68; *p* = 0.011; mean difference = 0.87; 95% CI: 0.21–1.54; Cohen's *d* = 0.41).

### 3.4 Awareness Level

#### 3.4.1 Overall Awareness Score

The mean awareness score was 14.45 ± 5.14 (range: 5–25). The scale showed high internal consistency (Cronbach's *α* = 0.88). All items had corrected item‐total correlation coefficients above 0.70, and removing any item did not increase Cronbach's alpha.

#### 3.4.2 Analysis of Awareness Items

Descriptive statistics for the awareness items are shown in Table 4. The highest mean score was observed in the item “I know what to do if a wire pierces the patient's cheek” (3.39 ± 1.31), while the lowest mean score was in the item “My training is sufficient to handle orthodontic emergencies” (2.47 ± 1.16).

**TABLE 4:** Mean scores for awareness items.

Item	Mean ± SD
I know what to do if a wire pokes into the patient's cheek.	3.39 ± 1.31
I can provide initial management recommendations for pain caused by orthodontic appliances.	3.11 ± 1.23
I have sufficient knowledge to identify orthodontic emergencies.	2.82 ± 1.10
I know how to manage a bracket detachment in an emergency situation.	2.66 ± 1.31
My training is sufficient to manage orthodontic emergencies.	2.47 ± 1.16

*Note:* Responses were measured on a 5‐point Likert scale (1 = strongly disagree, 5 = strongly agree).

### 3.5 Relationship Between Knowledge and Awareness

A statistically significant, positive, and low‐to‐moderate correlation was observed between the total knowledge score and the awareness score (Spearman *ρ* = 0.295; *p* < 0.001).

When assessing the relationship between awareness items and the knowledge score, all items were found to be statistically significant and positively associated with the knowledge level (*p* < 0.001 for all comparisons). The strongest association was observed in the intervention item (Spearman *ρ* = 0.308), while the weakest was seen in the educational adequacy item (*ρ* = 0.208).

A low positive correlation was observed between the knowledge score and the length of professional experience across the entire sample (*ρ* = 0.185; *p* < 0.001). However, when students were excluded and only graduate dentists were analysed, this correlation was not statistically significant (*ρ* = −0.104; *p* = 0.153).

The correlation analysis results are shown in Table 5.

**TABLE 5:** Correlation analysis between knowledge score and selected variables.

Variable	Spearman‘s *ρ*	*p*
Awareness score	0.295	< 0.001
Experience (entire sample)	0.185	< 0.001
Experience (graduates)	−0.104	0.153

Abbreviation: *ρ*: Spearman's rank correlation coefficient.

### 3.6 Multiple Linear Regression Analysis

In the multiple linear regression analysis conducted to identify factors independently associated with knowledge level, the model was found to be statistically significant (*F*(4.455) = 19.96; *p* < 0.001). The model explains 14.9% of the variance in the knowledge score (*R* ^2^ = 0.149; adjusted *R* ^2^ = 0.142).

In the adjusted regression model, awareness score showed the strongest association with knowledge score (*β* = 0.302; *p* < 0.001). In terms of occupational status, general dentists (*B* = 0.453; 95% CI: 0.01–0.89; *p* = 0.046) and specialists (*B* = 0.717; 95% CI: 0.02–1.41; *p* = 0.044) had significantly higher knowledge scores than students. Regarding the gender variable, male participants were found to have significantly lower knowledge scores than females (*B* = −0.77; 95% CI: −1.19 to −0.35; *p* < 0.001).

No multicollinearity issue was detected in the model (VIF < 1.2 for all variables).

Detailed results of the regression analysis are shown in Table 6.

**TABLE 6:** Results of multiple linear regression analysis regarding factors independently associated with knowledge level.

Variable	*B*	SE	*β*	95% CI	*p*	VIF
Constant	5.975	0.418	—	5.15–6.79	< 0.001	—
General dentist	0.453	0.227	0.091	0.01–0.89	0.046	1.12
Specialist	0.717	0.356	0.091	0.02–1.41	0.044	1.10
Awareness score	0.135	0.020	0.302	0.10–0.17	< 0.001	1.06
Male	−0.772	0.211	−0.162	−1.19 to −0.35	< 0.001	1.04

*Note:* Model statistics: *R* ^2^ = 0.149; adjusted *R* ^2^ = 0.142; *F* (4,455) = 19.96; *p* < 0.001. Reference categories: student (occupational status) and female (gender).

Abbreviations: *B*, unstandardized regression coefficient; CI, confidence interval; SE, standard error; VIF, variance inflation factor; *β*, standardized regression coefficient.

## 4 Discussion

This study is among the most comprehensive evaluations of the knowledge and awareness levels of dental students and graduate dentists in Türkiye regarding orthodontic emergencies, both collectively and at the cognitive level. While it is well known in the international literature that the level of knowledge among general dentists regarding orthodontic emergencies varies significantly across countries [8], the vast majority of existing studies are based on a single educational institution and do not simultaneously address student‐graduate comparisons and cognitive‐level analysis [9, 10]. With its multi‐center sampling structure and cognitive classification framework, this study aims to fill the methodological gap mentioned above; its findings are considered to be of reference value not only in the Turkish context but also in international discussions regarding the structuring of dental curricula.

Cognitive performance analysis showed that participants achieved high success in basic clinical management questions (92.2%), but their performance dropped to 58% in clinical prioritization and emergency intervention tasks. This aligns with studies indicating that physicians are skilled in technical procedures but may struggle with triage and clinical reasoning [11]. When the items were classified as conceptual, basic clinical management, and clinical prioritization within Bloom's cognitive taxonomy, the observed performance pattern may suggest that participants performed better on questions reflecting lower‐order cognitive processes than on those requiring higher‐order skills such as analysis and evaluation. However, the present study did not directly evaluate the effectiveness of the training curriculum. Clinical prioritization involves not only knowing the correct information but also interpreting it in context, performing risk assessments, and making decisions under time constraints [7]. These findings may suggest that further curricular evaluation is warranted, particularly regarding clinical prioritization and emergency decision‐making. Scenario‐based learning approaches may be considered as evidence‐informed options for future curriculum development rather than as direct implications of the present findings.

Performance analysis based on questions shows that knowledge about the definition of orthodontic emergencies is somewhat adequate, but some basic concepts are not fully internalized. While it is encouraging that most participants correctly identified that gum bleeding should not be considered an orthodontic emergency, it is notable that 41.7% failed to recognize situations like retainer breakage, wire penetration, and bracket detachment as emergencies. This indicates that understanding and recognition of what constitutes an “orthodontic emergency” in a clinical setting need improvement. The fact that the vast majority knew that antibiotic use is not indicated for pain management reflects a positive understanding of basic pharmacological principles.

However, a partial tendency to choose Nonsteroidal Anti‐Inflammatory Drugs (NSAIDs) over paracetamol as the first‐line pain reliever was observed. Animal and clinical studies have shown that NSAIDs can slow down orthodontic tooth movement by inhibiting prostaglandin synthesis; therefore, paracetamol has become the preferred agent during active treatment [12, 13]. Conversely, the clinical significance of short‐term NSAID use at clinical doses on tooth movement remains debated [14]. Although 75.7% of participants identified paracetamol as the correct answer, indicating fairly high awareness, only 10.9% preferred ibuprofen, which suggests that this clinical nuance has not yet been fully adopted in practice. This highlights that choosing analgesics should consider not only symptom relief but also the biological dynamics of the treatment. It seems that the distinction between acute intervention and definitive treatment in cases of retainer breakage and wire penetration is not fully understood. The fact that 23.3% of participants recommended making a new retainer as the primary response to retainer breakage, and 30.0% favored direct wire cutting over temporary relief methods for wire penetration, suggests confusion between symptom control and permanent treatment planning.

However, the primary goal in emergency management is to control pain and trauma; definitive planning should be assessed in the second stage [15]. These response patterns may indicate that the distinction between acute symptom relief and definitive treatment planning was not consistently recognized by all participants. This observation may support further evaluation of whether structured emergency‐management algorithms could be useful in future educational initiatives [16, 17, 18]. Similar response patterns in more specific complications, such as mini‐screw loosening and a Transpalatal Arch (TPA) embedded in the palate, may suggest areas in which further educational evaluation could be considered, particularly regarding recognition of situations requiring urgent referral or intervention [19].

Graduate dentists had higher knowledge scores than students. In this cross‐sectional sample, this difference was associated with occupational status and may be consistent with the potential contribution of greater clinical exposure. However, the present design does not permit conclusions regarding whether clinical exposure directly improves knowledge levels [9]. Among graduate participants, duration of professional experience was not significantly associated with knowledge score. This finding should be interpreted cautiously, as the study did not assess other dimensions of competence or continuing professional development [20]. Similarly, fifth‐year students had higher knowledge scores than fourth‐year students. This finding may reflect differences in educational stage, clinical exposure, or other unmeasured characteristics; however, the cross‐sectional design does not allow the temporal or causal contribution of these factors to be determined [9].

In the regression analysis, awareness score was the variable most strongly associated with knowledge score. Although this finding may be discussed in relation to Bandura's self‐efficacy theory, which conceptualizes self‐efficacy as an individual's belief in their capability to perform a specific task [3, 4, 5], the scale used in the present study assessed awareness and perceived competence rather than clinical self‐efficacy using a validated self‐efficacy instrument. Therefore, this theoretical framework should be considered a plausible interpretation rather than a mechanism directly supported by the study findings. Future studies employing validated clinical self‐efficacy instruments, together with longitudinal or intervention‐based designs, are needed to clarify the relationship between perceived competence, self‐efficacy, knowledge, and clinical decision‐making in orthodontic emergencies.

The positive association between awareness score and knowledge score suggests that participants reporting greater perceived competence also tended to have higher knowledge scores. However, this relationship should not be interpreted as evidence that perceived competence determines knowledge level, because the direction of the association and the influence of potential confounding factors cannot be established in a cross‐sectional study. The relatively low score for the item “My training is sufficient to manage orthodontic emergencies” may indicate that participants perceived limitations in their preparedness for these situations.

Previous literature suggests that undergraduate orthodontic curricula vary substantially in their content, assessment methods, and opportunities for clinical exposure [10, 21]. In light of the present findings, educational approaches aimed at improving clinical preparedness, such as competency‐based teaching and supervised simulation activities, may be considered as evidence‐informed future directions. However, the effectiveness of these approaches for improving awareness, knowledge, self‐efficacy, or clinical performance in orthodontic emergencies should be evaluated in prospective intervention studies.

The regression model explained 14.9% of the variance in knowledge scores (*R* ^2^ = 0.149; adjusted *R* ^2^ = 0.142). Although the model was statistically significant, approximately 85% of the variability in knowledge scores remained unexplained, indicating that its predictive capacity was limited. Knowledge regarding orthodontic emergencies may be influenced by a range of unmeasured educational, clinical, individual, and institutional factors, including prior orthodontic training, the quality and extent of clinical exposure, curriculum characteristics, access to continuing professional education, individual motivation, and previous experience with orthodontic emergencies [22]. Accordingly, awareness score, occupational status, and gender should be interpreted as variables associated with knowledge score within this sample rather than as a comprehensive explanation of knowledge level. These findings may help identify areas for further investigation, but they should be interpreted cautiously given the substantial proportion of unexplained variance. Future studies incorporating a broader range of educational, clinical, and institutional variables may provide a more complete understanding of factors associated with knowledge regarding orthodontic emergencies.

While it is noteworthy that female participants showed higher knowledge scores, caution should be exercised when interpreting this difference. Although literature reports that female health professionals emphasize protective practices and protocol adherence more, this trend is not universal and is affected by cultural and institutional factors [23, 24, 25]. Since potential confounders such as education type, clinical exposure, and areas of interest were not systematically controlled for in this study based on gender, it is inappropriate to attribute the observed difference directly to gender; this finding should be considered a variable to be controlled for in future research.

Participants who reported actively providing orthodontic treatment had higher knowledge scores than those who did not. This association may reflect greater exposure to orthodontic cases, prior training, professional interest, or other unmeasured factors. Because of the cross‐sectional design, it cannot be concluded that clinical practice itself led to higher knowledge scores [8, 26]. The more frequent preference for removable appliances among practitioners suggests that general dentists prioritize interventions with lower complication risks and refer complex cases to orthodontic specialists [27]. These considerations may support further evaluation of how general dentists can be prepared to provide initial management of basic orthodontic emergencies, particularly in settings where access to orthodontic specialists may be limited.

This study has several limitations that should be considered when interpreting the findings. First, convenience sampling was used, and approximately half of the participants were students or graduates of a single university. Therefore, the sample may not fully represent dental students, general dentists, or specialists across Türkiye. Differences in institutional curricula, clinical training opportunities, regional distribution of orthodontic services, and access to continuing professional education may limit the external validity and generalizability of the findings to other educational settings or professional populations.

Second, data were collected through an online self‐report questionnaire. This approach may have introduced selection bias, as participants with greater interest in orthodontics or stronger motivation to complete the survey may have been more likely to participate. Response bias, recall bias, and the possibility that participants consulted external sources while completing the questionnaire also cannot be excluded. In addition, the cross‐sectional design does not allow causal or temporal interpretations of the observed associations.

Future studies should employ more representative sampling strategies, such as stratified or cluster sampling across multiple universities and geographical regions. Recruitment of participants from public, private, and university‐based dental settings may also improve the representativeness of graduate dentists. Multi‐center studies with balanced representation across educational institutions, professional groups, and regions would strengthen the external validity of future research.

The observed gaps in clinical prioritization and decision‐making suggest that orthodontic emergency education may warrant further curricular evaluation. Although simulation‐based training, case‐based learning, and Objective Structured Clinical Examinations (OSCEs) style assessments were not evaluated in the present study, these approaches have been supported in the broader dental and health professions education literature as methods that may enhance clinical reasoning, prioritization, and procedural preparedness [28, 29, 30].

Accordingly, simulation activities, case‐based scenarios, and OSCE‐style assessments may be considered as evidence‐informed educational proposals for future curriculum development. However, their effectiveness in improving knowledge, awareness, clinical reasoning, or emergency‐management performance in orthodontic emergencies should be assessed through prospective and intervention‐based studies.

## 5 Conclusions

In conclusion, although dentists and dental students have basic knowledge of managing orthodontic emergencies, they show significant gaps in clinical prioritization and decision‐making skills. Participants reported relatively low perceived adequacy of training for managing orthodontic emergencies. Together with the observed gaps in clinical prioritization, this finding may identify an area for further curricular evaluation. Prospective and intervention‐based studies are needed to determine whether specific educational approaches improve knowledge, clinical reasoning, or emergency‐management performance.

The findings may help identify areas for future curricular evaluation, particularly with respect to clinical prioritization and emergency decision‐making. Based on the broader educational literature, scenario‐based learning, simulation activities, and OSCE‐style assessments may be considered as evidence‐informed approaches for future curriculum development. However, because these educational interventions were not directly evaluated in the present study, their effectiveness should be examined in prospective and controlled educational studies. Future research may also evaluate the role of continuing professional development in improving preparedness for orthodontic emergencies.

## Author Contributions

K.A.C. contributed to the conception and design of the study, development of the questionnaire, data collection and analysis, and drafted the manuscript. T.Y. contributed to the literature review, questionnaire development, and critical revision of the manuscript. All authors read and approved the final manuscript.

## Funding

The authors have nothing to report.

## Ethics Statement

The study was approved by the Mersin University Clinical Research Ethics Committee (decision no. 2025/1079; October 1, 2025). Informed consent was obtained electronically from all participants prior to participation.

## Consent

The authors have nothing to report.

## Conflicts of Interest

The authors declare no conflicts of interest.

## Data Availability

The datasets used and/or analysed during the current study are available from the corresponding author on reasonable request.
